# Building Block-Centric Approach to DNA-Encoded Library Design

**DOI:** 10.1021/acs.jcim.4c00232

**Published:** 2024-06-11

**Authors:** Patrick R. Fitzgerald, Anjali Dixit, Chris Zhang, David L. Mobley, Brian M. Paegel

**Affiliations:** † Skaggs Doctoral Program in the Chemical and Biological Sciences, Scripps Research, La Jolla, California 92037, United States; ‡ Department of Pharmaceutical Sciences, University of California, Irvine, California 92697, United States; § Department of Chemistry, University of California, Irvine, California 92697, United States

## Abstract

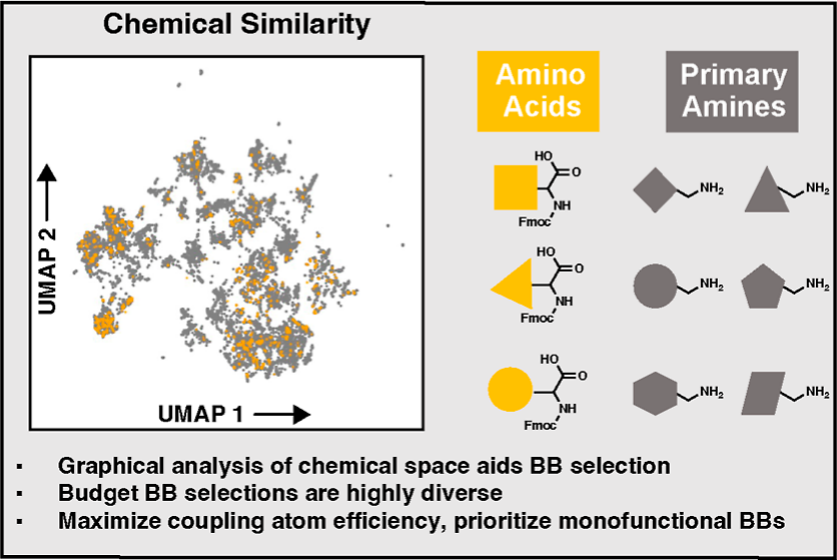

DNA-encoded library technology grants access to nearly infinite opportunities to explore the chemical structure space for drug discovery. Successful navigation depends on the design and synthesis of libraries with appropriate physicochemical properties (PCPs) and structural diversity while aligning with practical considerations. To this end, we analyze combinatorial library design constraints including the number of chemistry cycles, bond construction strategies, and building block (BB) class selection in pursuit of ideal library designs. We compare two-cycle library designs (amino acid + carboxylic acid, primary amine + carboxylic acid) in the context of PCPs and chemical space coverage, given different BB selection strategies and constraints. We find that broad availability of amines and acids is essential for enabling the widest exploration of chemical space. Surprisingly, cost is not a driving factor, and virtually, the same chemical space can be explored with “budget” BBs.

## 1 Introduction

DNA-encoded library (DEL) synthesis grants nearly limitless access to a large and novel chemical space—the raw materials of early drug discovery. DELs assign unique DNA barcodes to all possible combinations of small-molecule building blocks (BBs) during encoded split-and-pool combinatorial synthesis.^1^ Then, by leveraging the massive throughput of genome sequencers, DELs are analyzed by affinity selection to identify all BB combinations that produce a ligand of the target protein. Numerically large libraries can be rapidly synthesized and screened to furnish hits that occupy a distinct chemical space.^2,3^ DEL has identified novel small-molecule hits against many targets^4^ and yielded clinical candidates.^5−9^ These successes have driven a virtuous cycle of developing new capabilities in library design, synthesis, and analysis.^10^

Library design is a primary factor affecting the likelihood that DEL hits will lead to a development candidate. A successful DEL campaign will produce several hit series with attractive physicochemical properties (PCPs). As an example, Pfizer employs a DEL hit scoring metric that is highly influenced by molecular weight (MW), wherein >600 Da hits are ignored.^11^ PCPs tend to inflate undesirably as the number of chemistry cycles increases in a combinatorial library synthesis.^12,13^ DEL reaction development efforts have aimed to achieve robust, atom-efficient coupling, but the rate of DEL reaction development has seemingly outpaced the rate of implementation in library synthesis and selection.^14,15^ DEL reaction development efforts often focus on synthetic yield and DNA compatibility,^16−18^ which are critical features to consider during DEL synthesis. However, additional aspects, such as compatible BB availability, reaction scheme simplicity, and library member PCP space are not widely discussed. These considerations are critical to effective combinatorial library design, having figured prominently in GSK’s retrosynthetic combinatorial analysis procedure.^19^

Early DEL reaction sequences provided critical insight for improving DEL design. These library designs,^1,6−8,20^ which featured numerous cycles of chemistry (three to four), skewed library PCPs away from the attractive rule-of-5 (RO5)^21^ space that is preferred as a starting point for lead optimization.^12^ Additionally, use of a core scaffold increased MW and hydrophobicity while confining the library to a specific region of chemical space.^1,20,22^ Libraries from this era successfully identified leads that developed into clinical candidates against RIP1^5,6^ and sEH,^7,8^ but both clinical candidates are truncates from three and four-cycle libraries, respectively. Identifying active truncates is a challenge that has been addressed with the development of on-DNA hit validation methods^23−26^ but still requires significant downstream investment in hit evaluation.^10^ However, looking for truncates in libraries with many (>3) cycles of chemistry is a low signal-to-noise proposition: library size increases exponentially with the number of chemistry cycles,^12^ leading to increased false-negative rates.^27^ Therefore, screening a DEL with fewer coupling steps is likely advantageous.^22^

In this work, we specify the principles for building DELs that maximize the likelihood of yielding hits with desirable PCPs for future development. These principles are consistent with our previous assertion that DEL technology, like “click chemistry," should explore easily accessible regions of chemical space.^18^ Expanding upon this concept, DEL should prioritize reactions that use widely available BB sets. To demonstrate, we visualize BB coverage of chemical space using a dimensionality reduction technique, uniform manifold approximation and projection (UMAP).^28^ From UMAP projections of commonly used BB sets, we demonstrate the effect that various BB selection strategies have on the properties of final output libraries. Collectively, these analyses provide a framework for the computational design and characterization of BBs for DEL synthesis.

## 2 Results and Discussion

### 2.1 Library Design

**Figure 1:**
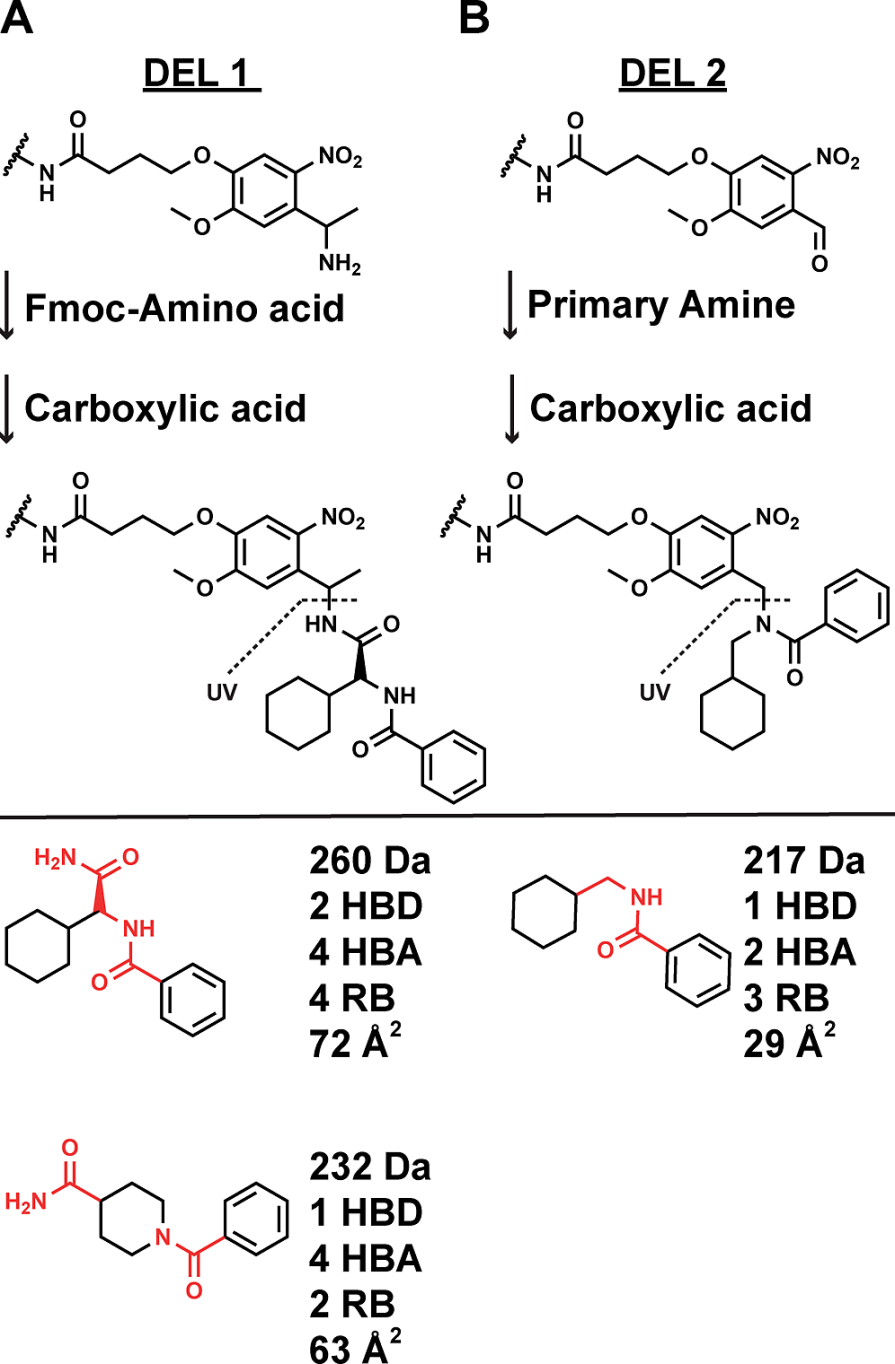
Property-focused analysis of DEL synthesis strategies. (A) Synthesis of DEL1 proceeds on an amine-functionalized photocleavable linker. Fmoc–AA coupling occurs in the first chemistry cycle, followed by carboxylic acid coupling in the second step. Compounds photocleave at the indicated bond (UV) as primary amides. Primary amine and secondary amine containing Fmoc–AAs are depicted along with their calculated properties. The constant library scaffold is indicated (red). (B) DEL2 synthesis starts from an aldehyde-functionalized photocleavable linker. Primary amines are coupled by reductive amination in the first chemistry cycle, followed by carboxylic acid coupling in the second step. Compounds photocleave at the indicated bond (UV) as secondary amides. An example compound is illustrated along with its calculated properties according to RO5 criteria.

To illustrate our library design principles, we compare a previously employed two-cycle library design with a plausible alternative two-cycle library design. In our previous library design, DEL1,^29−31^ an amine-functionalized *o*-nitroveratryl photocleavable linker, was acylated with Fmoc–amino acids (Fmoc–AAs) in the first chemistry cycle, then the pendant Fmoc was removed, and carboxylic acids were coupled in the second cycle (Figure 1A). DEL1 required four synthetic manipulations, and each library compound photocleaved from beads possessed a pendant primary amide and a BB-connecting amide. In the proposed alternative design, DEL2, an aldehyde-functionalized photocleavable linker is coupled with primary amines in the first cycle through reductive amination, and the resulting secondary amine is acylated with carboxylic acids in the second cycle (Figure 1B). When liberated from the bead, library products contain only a BB-connecting amide.^32^ The previous library design effectively contributes an extra –CONH_2_ from the photocleavage product, which adds two hydrogen bond acceptors, one hydrogen bond donor, and 44 Da.

The DEL2 synthesis scheme offers several advantages over the DEL1 scheme. DEL2 requires fewer synthetic manipulations during library synthesis, introduces less bond construction “baggage,” and is overall more atom efficient. Additionally, hits from DEL2 can be synthesized in solution from commercially available materials in a single step, allowing for facile exploration of additional derivatives.^33^ DEL2 is likely to be productive against many targets as amides are the most common functional group in bioactive compounds,^34^ and their formation is the most prominent reaction in medicinal chemistry hit optimization campaigns.^35^ Previous DEL selections highlight the utility of libraries like DEL2.^36−39^

### 2.2 Building Block UMAP Comparison

We posited that by tapping amines, a substantially larger BB pool than Fmoc–AAs, DEL2 would sample additional valuable chemotypes. To assess the diversity of the BB pools used in DEL1 and DEL2, we analyzed Enamine’s commercial Fmoc–AA, primary amine, and carboxylic acid collections using UMAP. We refer to these collections as BB sets. First, we truncated BB sets by the replacement of defining functional groups (NH_2_, COOH, and *N*-Fmoc) with –H (Table S1). Next, we calculated Morgan ECFP6 fingerprints (radius = 3) for each truncate structure and used the fingerprint to generate a 2D-Tanimoto similarity matrix for all BB truncates. Finally, we employed UMAP to convert a high-dimensional representation of the similarity matrix to a 2D visualization. As the similarity matrix was generated from all three BB sets, the projection of chemical space was the same for each set, though the projected UMAP distances may not directly correlate with Tanimoto similarity scoring difference. Implementing our UMAP analysis without first truncating the BB sets yielded higher Tanimoto similarity scores and more densely populated UMAP plots, particularly for carboxylic acid and Fmoc–AA BBs (Figure S1).

**Figure 2:**
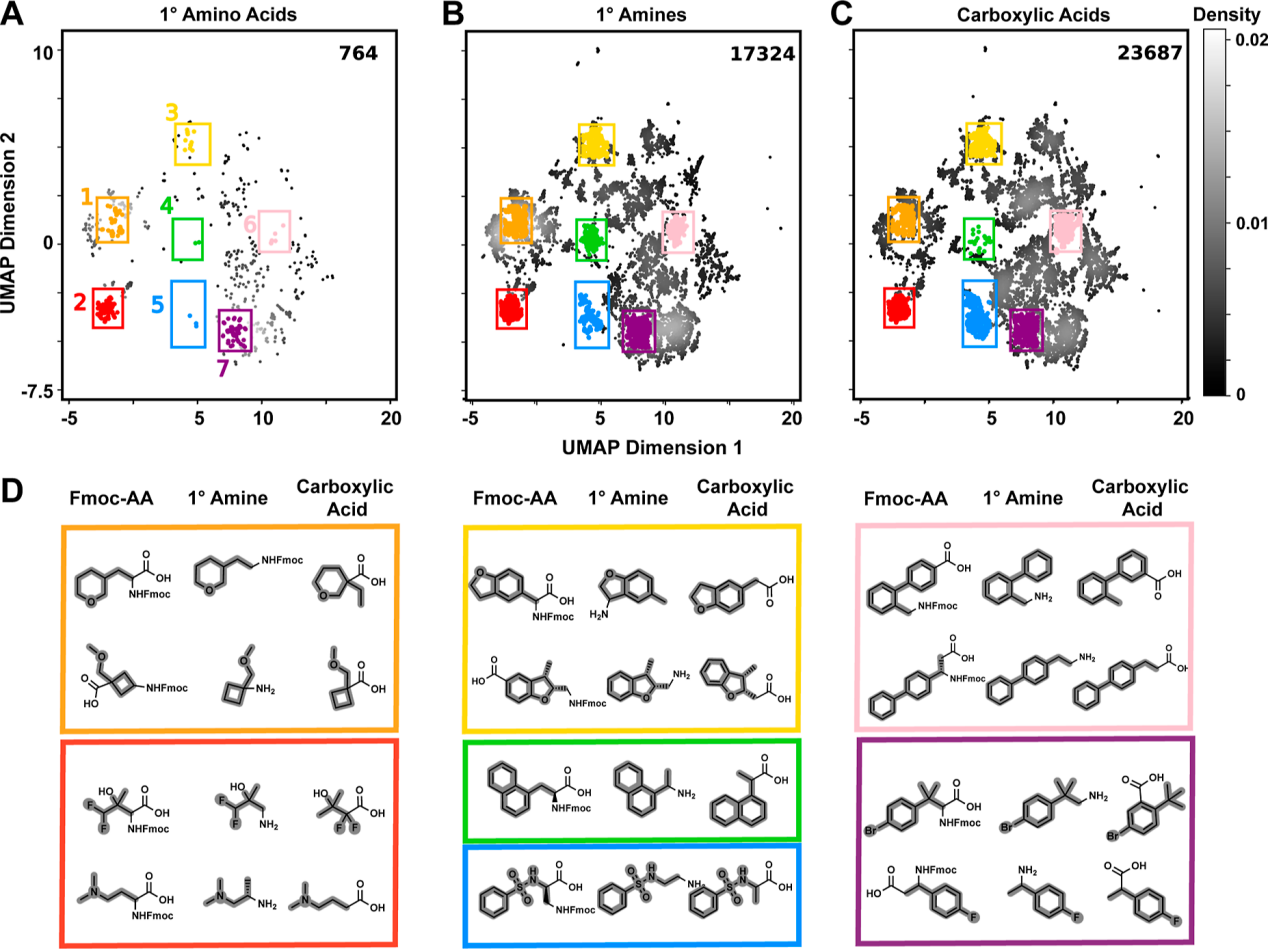
UMAP analysis of Fmoc–amino acid, primary amine, and carboxylic acid BBs from Enamine as truncates. Density plots arranged by chemical similarity are compared for (A) Fmoc–amino acid, (B) primary amine, and (C) carboxylic acid truncates. Grayscale intensity denotes the probability density of points. BB truncate counts in each pool are indicated in the top right of each plot. (D) Example BBs from Fmoc–AA, primary amine, and carboxylic acid sets are depicted for specific regions of UMAP space [colored points/rectangular outlines in (A–C)], and their truncates are indicated (bold). Fmoc–AAs cover a limited portion of the UMAP space relative to primary amines or carboxylic acids.

Fmoc–AAs, the smallest BB set, covered the narrowest area of chemical space (Figure 2A). Primary amines were far more numerous, and their truncates covered the areas of UMAP space occupied by Fmoc–AA truncates while densely populating previously unpopulated regions (Figure 2B). Additionally, when analyzed by direct comparison of truncate structures, very few (*n* = 78) truncate structures were unique from the Fmoc–AA set (Figure S2). Carboxylic acids, upon truncation, covered similar areas of chemical space as both Fmoc–AA and primary amine truncates (Figure 2C). The increased coverage observed for amines and carboxylic acids relative to that for amino acids appeared to be a reflection of increased breadth as well as depth (Figure S3). UMAP analysis readily identified the most prevalent chemotypes, supporting the assumption that distance on the UMAP plot corresponded to chemical similarity (Figure 2D). As examples of overlapping chemotype representation, all three BB sets thoroughly sampled linear aliphatics, cycloalkanes, and substituted benzene chemotypes (regions 1, 2, and 7, respectively). On the other hand, regions represented by 2,3-dihydrobenzofuran, naphthalene, benzylsulfonamide, and biphenyl chemotypes (regions 3, 4, 5, and 6, respectively) are poorly represented by the Fmoc–AA set while being well represented by the primary amine and carboxylic acid BB sets.

The large enhancement in the chemical space coverage of primary amine or carboxylic acid BB sets is a prime advantage of using monofunctional BB sets. Previous analysis established the difference in the quantity of these and other BB classes,^40^ yet a direct comparison of their chemical similarity has not been conducted. Our analysis hinted at structural limitations resulting from designing a DEL that includes Fmoc–AAs. Namely, such a library is likely to be biased toward linear aliphatic, cyclic aliphatic, and benzene derivatives as the three main chemotypes. Notably, inhibitors of sEH and Wip1 derived from DEL screening both feature cycloalkane Fmoc–AA BBs.^8,41^ The BB chemotypes that are more highly represented in amine and carboxylic acid sets represent valuable materials for exploring chemical space. The substituted dihydrobenzofuran moiety is a key pharmacophore in a potent bromodomain 2 inhibitor.^42,43^ Naphthyl groups feature in inhibitors of ADAMTS5,^44^ SARS-CoV-2 PLpro,^45^ and SARS CoV2 mPro.^46^ Sulfonamides are found in multiple approved drugs. Finally, biphenyls are of clear interest to DEL practitioners, given the heavy investment in DNA-compatible Suzuki reaction development.^20,31^ These observations in concert with library selection analyses^47^ and recent modeling experiments^3^ indicate that it is advantageous to design DELs that draw from deep commercial pools.

### 2.3 BB Diversity as a Function of BB Cost

**Figure 3:**
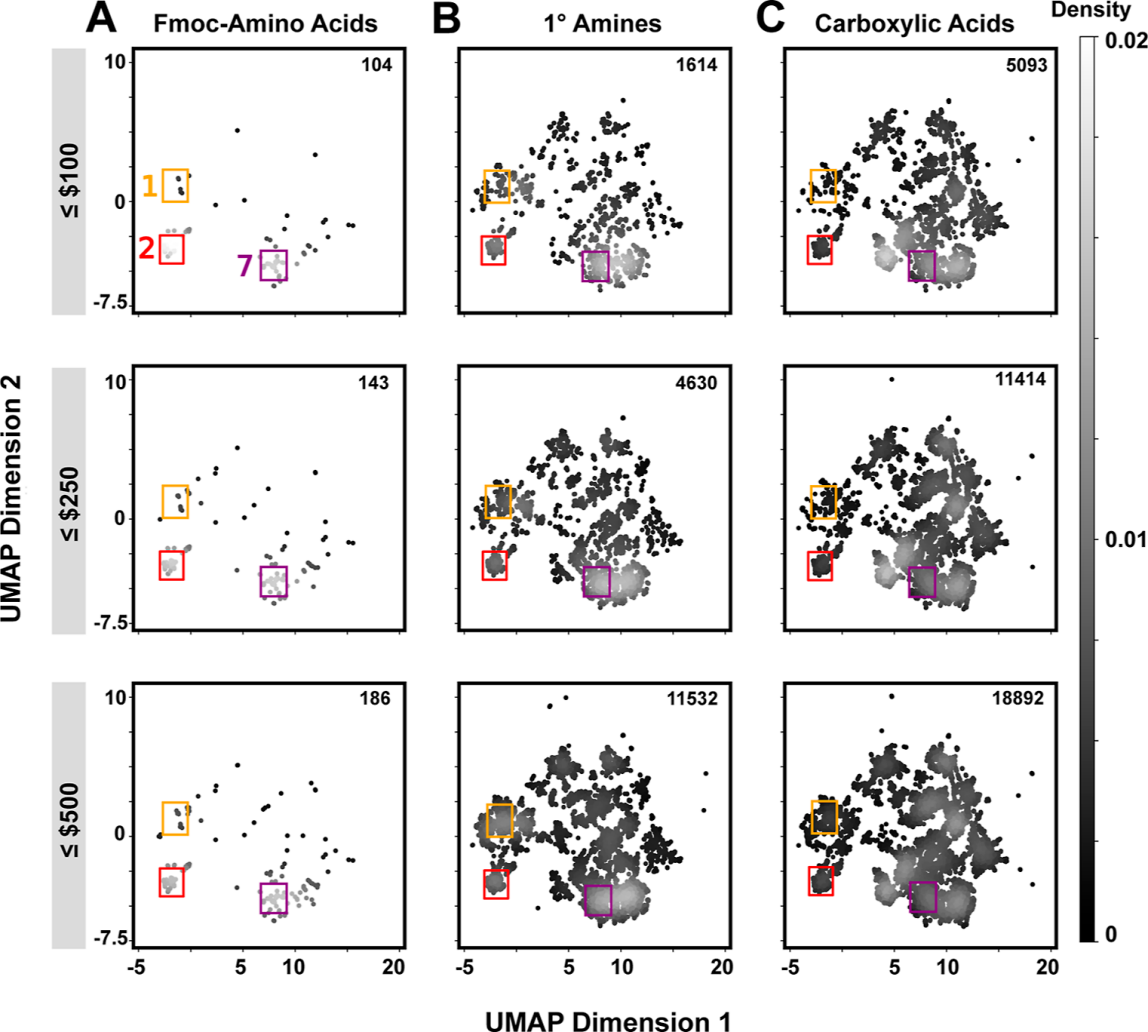
Availability of BB truncates following cost filtering. UMAP analyses are shown for three different cost thresholds (≤$100, ≤$250, and ≤$500 per 250 mg) for (A) Fmoc–AAs, (B) primary amines, and (C) carboxylic acids. Grayscale intensity denotes the probability density of points. BB truncate counts in each pool are indicated on the top right of each plot. Primary amines and carboxylic acids cover more diverse chemical space compared to Fmoc–amino acids at all cost points, especially at the lowest cost cutoff.

We next sought to understand how BB cost constraints might influence the accessibility of diverse chemotypes. The cost of Fmoc–AA, primary amine, and carboxylic acid BB sets all conform to roughly bimodal distributions (Figure S4). We stratified BBs by applying cost cutoffs (≤$100, ≤ $250, and ≤$500 per 250 mg) and then generated corresponding UMAP plots (Figure 3) and Venn diagrams (Figure S5). While the density of UMAP plots decreases for all three BB classes with stricter cost filtering, cost most influences Fmoc–AAs (Figure 3A). The two most represented chemotypes within Fmoc–AAs—linear aliphatics (region **2**) and substituted benzenes (region **7**)—remain accessible at the lowest cost filter, but most other regions do not. The third most common grouping, cycloalkanes (region **1**), are only accessed above $500/250 mg. For primary amines, the majority of UMAP space remains accessible even at the lowest price cutoff, albeit slightly more sparsely (Figure 3B). Additionally, upon comparison of identical truncate scaffolds (Figure S6), amines are substantially cheaper than their Fmoc–AA counterparts (median cost difference for 250 mg = $509). Even after stringent cost filtering, the carboxylic acid set included in both DEL1 and DEL2 is the most abundant BB class and broadly covers the UMAP space (Figure 3C). At all the selected cost cutoffs, primary amines and carboxylic acids more thoroughly sample the UMAP space compared to Fmoc–AAs.

Our analysis emphasizes the importance of designing library reaction sequences that can tap large and economical BB pools while also minimizing the atoms involved in bond construction and simplifying the interpretation of synthesis products. Library synthesis and hit synthesis budgets may vary drastically between different DEL practitioners, but understanding the effect of cost on potential library outcomes is universally important. Even under generous cost constraints (<$500/250 mg), the Fmoc–AA set samples only 186 truncates and cost-limited Fmoc–AAs even more sparsely sample chemical space. Although our analysis was limited to Fmoc–AAs, primary amines, and carboxylic acids available from one vendor, these BB classes are among the most common in DEL synthesis.^18^ More generally, monofunctional BB classes tend to have more members than bifunctional BB classes,^13^ so the trends observed here are likely general. Implementing economical BB classes with robust and broad “click-like” scope^18^ also eases the prioritization of hit synthesis and validation, a major bottleneck for DEL technology.^23−26^ Looking forward, these simplified library designs incorporating economical BBs further position DEL screening output to feed into direct-to-biology platforms for higher throughput structure exploration and lead optimization via cellular activity assays.^48−50^

### 2.4 BB Diversity as a Function of Molecular Weight

**Figure 4:**
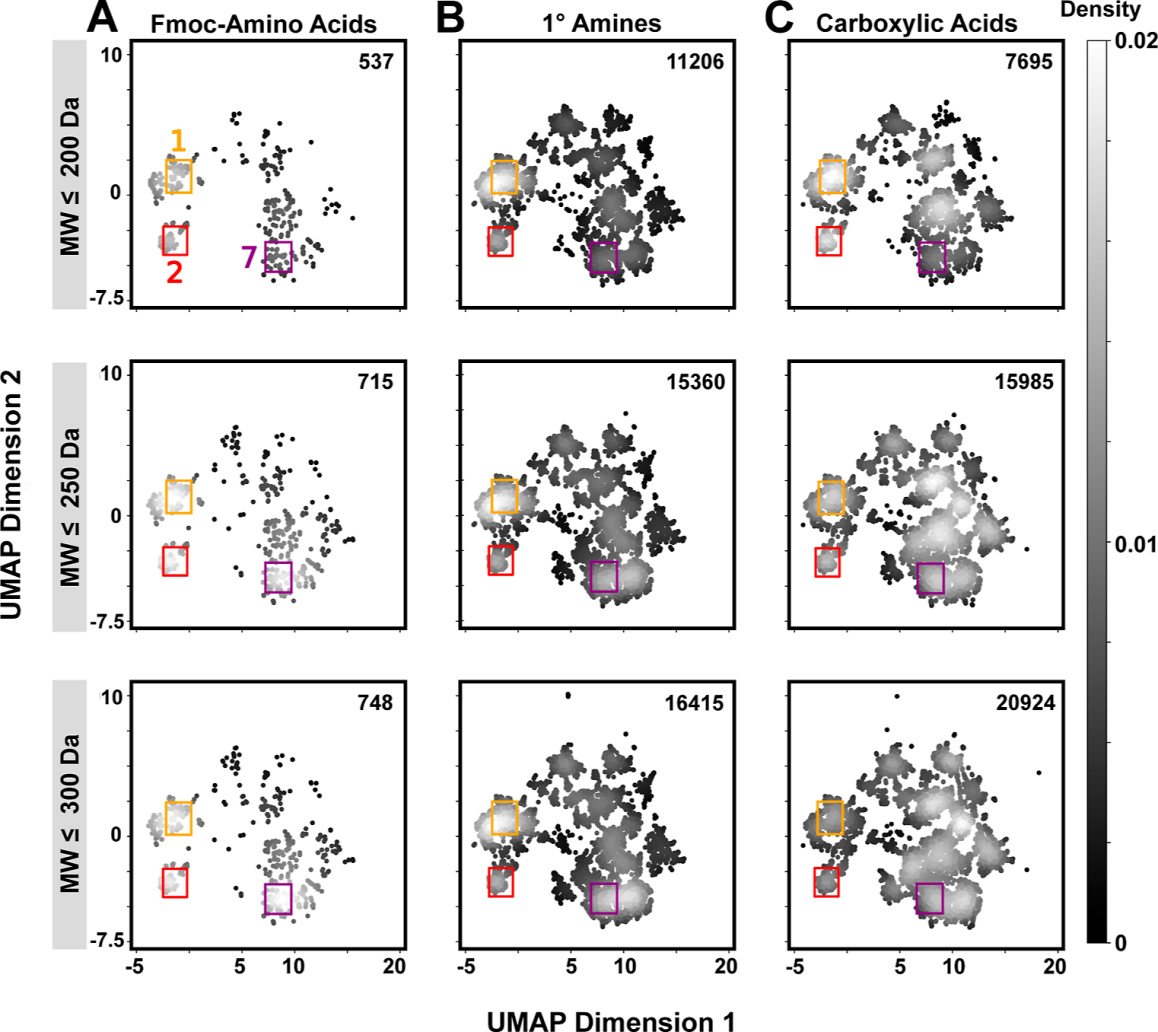
Availability of BB truncates following MW filtering. UMAP analyses are shown for three different MW thresholds (≤200, ≤250, and ≤300 Da) for (A) Fmoc–AAs, (B) primary amines, and (C) carboxylic acids. Grayscale intensity denotes the probability density. BB truncate counts in each pool are indicated on the top right of each plot. Filtering at these MW thresholds does not substantially alter the accessibility of chemical diversity for any of the BB sets. These regions (color coded) are shown to aid in comparison of identical UMAP regions between BB sets and MW filters.

We suspected that PCP filtering—a common aspect of DEL workflows—would also influence library structural diversity to different extents based on numerical BB set size. Fmoc–AA (ignoring Fmoc), primary amine, and carboxylic acid BB sets have similar MW but different clog *P* distributions [median MW = 171, 174, and 220 Da, and median clog *P* = −1.8, 0.69, and 1.1, respectively (Figure S7)]. To analyze the relationship between MW and chemical space, we apply three MW cutoffs (≤200, ≤250, and ≤300 Da) and visualize the resulting subsets using UMAP or Venn diagram (Figures 4 and S8). For the Fmoc–AAs, 70, 94, and 98% remain after filtering at ≤200, ≤250, and ≤300 Da cutoffs, respectively. Similarly, for primary amines, 65, 89, and 95% remain at the same cutoffs. Carboxylic acids experience a more drastic reduction, with 32, 67, and 88% remaining at these cutoffs. For all three sets, no specific regions are disproportionately affected by MW filtering. At all MW cutoffs, primary amine and carboxylic acid sets recapitulate the chemotypes present in the corresponding Fmoc–AA set while also offering numerous additional unique truncates.

UMAP analysis shows that MW filtering does not affect the availability of chemical diversity for any of the BB sets in this study. For the 2-cycle libraries DEL1 and DEL2, the ≤200, ≤250, and ≤300 Da MW cutoffs roughly correspond to library products within the druglike PCP RO5 constraints. These are important thresholds to consider when balancing chemical diversity against PCPs (e.g., MW). Previous analysis established that reasonable library PCPs are most readily obtained with optimized library design scheme rather than with strict BB filtering.^13^ These observations, along with the identification of high MW BBs in previously identified DEL hits have motivated some to prescribe including high MW BBs, though upper limits were not explicitly stated.^2,51^ Conversely, others have implemented BB filtering strategies to align their libraries with RO5 PCPs,^52^ and separately, medicinal chemists have adopted a “rule of 2” BB criteria during lead optimization.^53^ Our analysis supports implementing a MW cutoff during BB selection, as this does not appear to impact the sampling of chemical space. However, several important caveats bear consideration. First, our constraints focus on an analysis of 2-cycle libraries. Additional considerations likely play into BB election and scheme optimization for 3-cycle libraries. Second, the UMAP approach is a powerful tool for high-level visualization of chemical space, but may not capture more detailed features. Along these lines, truncates from a wide range of chemotypes remain represented at even the ≤200 Da cutoff, but these are less densely functionalized than their higher MW counterparts.

### 2.5 BB Diversity as a Function of the BB Selection Method

**Figure 5:**
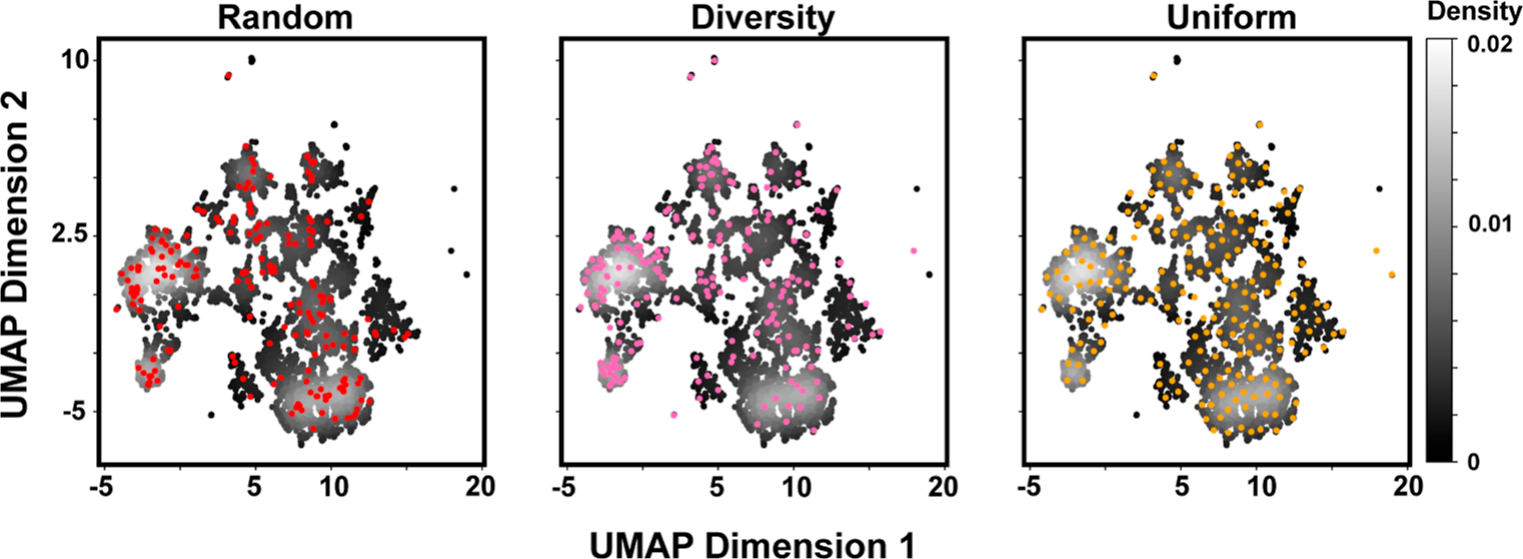
Impact of different BB selection strategies on chemical space sampling. Density plots of primary amine truncates arranged by chemical similarity are overlaid with BB selections (colored points) generated by random, diversity, and uniform methods. Grayscale intensity denotes the probability density of points. Random selection is biased toward dense UMAP regions. Diversity selection improves the sampling of sparsely populated regions but remains biased toward densely populated regions. Uniform selection maximizes the coverage of the UMAP space.

Having observed the effects of cost and property constraints on BB diversity, we sought to evaluate how different BB selection strategies impact chemical sampling. When sampled 192 times (2 × 96-well plates), the collection of amines containing ∼15,000 compounds offers an unfathomably large number of options . We explore three methods for sampling of BBs from very large pools: random, diversity, and uniform. We applied each strategy to select 192 BBs from the primary amine (Figure 5) and carboxylic acid pools (Figure S9). The random method selects 192 points from the collection of available BBs after all relevant filters have been applied. Random sampling selects more points from the most densely populated regions of chemical space, which follows trends in the commercial collection. The “diversity” method selects BBs with minimized nearest-neighbor Tanimoto similarity scores. We seed the selection with a randomly selected BB, find the most dissimilar BBs, and iterate until reaching 192 BB selections. UMAP analysis of diversity selection shows sampling of regions not covered by random selection, including singletons. Lastly, the “uniform” selection method uses the distances between points in the UMAP space rather than Tanimoto distance, minimizing sampling of densely populated regions of the UMAP space.

While it is tempting to prescribe uniform selection, since it maximizes UMAP coverage, each BB selection method raises distinct considerations. Random selection is biased toward the most abundant commercially available compound classes. Alternatively, strategic selection can increase the number of unique chemical fingerprints sampled while minimizing compound similarity.^54^ Minimizing similarity is not necessarily advantageous. The diversity method intentionally selects exotic and potentially undesirable compounds, such as silicates or perfluorocarbons. Selecting compounds from a similar compound class can be advantageous as certain regions of chemical space correlate with higher proclivity for protein binding. These “privileged” structures are frequently observed in drugs.^55^ The GSK benzimidazole scaffold is one such example specific from DEL.^56^ Similarity-based selection also biases against small changes to a scaffold, such as the introduction of a single methyl group. These subtle changes can profoundly alter the binding affinity.^57,58^ BB similarity across different positions (such as the inclusion of similar chemotypes in position 1 and position 2) is another design consideration. Completely overlapping chemotypes across both positions is likely unwise as this reduces overall library structural diversity. However, duplication of chemotypes across multiple positions could be beneficial for addressing symmetric protein targets^59^ or for increasing confidence in hit determination by observing similar chemotypes in multiple positions.^29^ Regardless of the selection strategy, high reaction yield should be a prerequisite for BB inclusion.^16,17^

### 2.6 Library Comparisons

**Figure 6:**
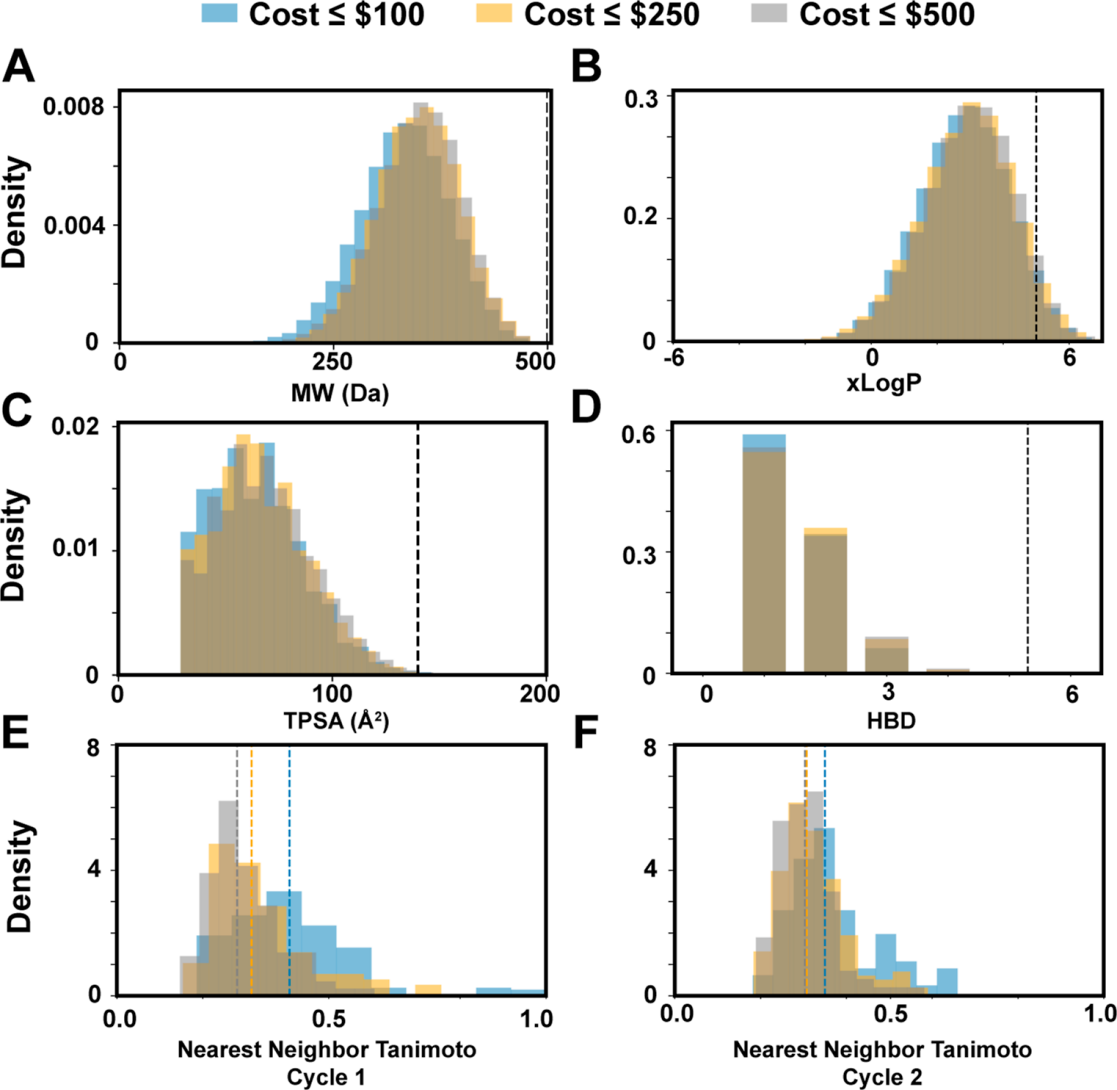
PCP and diversity summary statistics for three two-cycle libraries (condensation of 192 primary amines with 192 carboxylic acids) with different cost constraints. BBs are filtered by cost (≤$100, ≤$250, and ≤500 per 250 mg) and then randomly selected until reaching the intended split size (192). Libraries are enumerated, and their properties are compared. The PCPs of enumerated products including (A) MW, (B) *n*-octanol–water partition coefficient (*x* Log *P*), (C) total polar surface area, and (D) HBD count are calculated and plotted as density plots. Black dashed lines indicate RO5 thresholds. The complete similarity matrix of all BBs within a given library is calculated, from which nn-Tanimoto scores for (E) the primary amine BBs and (F) the carboxylic acid BBs are determined and plotted.

Having evaluated the effects of cost considerations, MW filtering, and selection method on BB diversity, we examine their combined effects during library design. We explore three 192 × 192 libraries following the DEL2 library design (primary amine + carboxylic acid) using a random BB picking strategy. These libraries shared the same PCP cutoffs (MW ≤ 250 Da) but differ in their cost constraints (≤$100, ≤$250, and ≤$500 per 250 mg). We enumerate all three libraries and calculate the PCPs of every library product. The distributions of MW, predicted *n*-octanol–water partitioning coefficients (*x* Log *P*), polar surface area, and hydrogen bond donor (HBD) count were nearly identical for all three libraries (Figure 6A–D). We use 2D nearest-neighbor Tanimoto scores to characterize intralibrary BB similarity as a measure of chemical diversity. Analyzing the primary amines (Figure 6E), the cheapest set has higher nearest-neighbor (nn) scores (median nn-Tanimoto_amines≤$100_ = 0.40) compared with the middle and highest cost sets, which were similar (median nn-Tanimoto_amines≤$250_ = 0.31 and median nn-Tanimoto_amines≤$500_ = 0.28). Analyzing the carboxylic acid set (Figure 6F), a similar trend was observed, with the lowest cost set having the highest nearest-neighbor (nn) scores (median nn-Tanimoto_COOH≤$100_ = 0.35) compared with the middle and highest cost sets (median nn-Tanimoto_COOH≤$250_ = 0.31 and median nn-Tanimoto_COOH≤$500_ = 0.30). These trends in nn-Tanimoto scores as a function of BB-cost were reproducible following replicate sampling (Figure S10).The total cost of BBs in each cycle scales proportionally to the maximum allowed BB cost in the library. For BB costs restricted at ≤$100, ≤$250, and ≤$500 per 250 mg, the total cost is roughly $10,000, $25,000, and $50,000, respectively, for both cycle 1 and cycle 2 BBs. We compared the enumerated library generated by random selection with a proportional sampling of an Enamine lead-like set and found that the DEL set had minimal chemical similarity to the lead-like set. Additionally, DEL members share higher nearest-neighbor Tanimoto similarity with other DEL members than lead-like compounds do with other lead-like compounds (Figure S11).

PCP distributions of enumerated libraries were minimally affected by cost filtering of BB sets, while intralibrary BB similarity followed a law of diminishing returns with respect to cost. These observations are specific to the split-size and BB sets examined but highlight important potential consequences of cost filtering. For large BB sets, cost filtering does not appear to impact PCP space accessibility as all three libraries shared similar PCPs. In contrast, BB diversity noticeably improved by increasing the cost filter from ≤$100 to ≤$250. However, continuing to increase the cost threshold to ≤$500 did not offer substantial improvement. Our comparison of the enumerated DEL with a comparative lead-like set illustrated important differences between DEL chemical space and compound screening collections. DEL compounds had higher intralibrary similarity, as expected from combinatorial library products featuring overlapping BBs. While ChEMBL has been used as a biologically active reference set to map DEL compounds,^3,40^ it is unclear how desirable the overlap of these chemical spaces should be because lead novelty is one of the technology’s primary advantages. DELs are orders of magnitude larger than HTS collections,^4^ DEL selection routinely identifies hits dissimilar from HTS screening sets,^5,6,36,47^ and DEL hits frequently access unexpected binding modes.^60^

### 2.7 Outlook

The BB-centric approach to the DEL design of this study ultimately aimed to understand ideality in the context of chemistry and BB selection. Our library design goals included minimizing nondiversity elements, limiting DELs to two to three couplings, leveraging numerically large BB sets, and prioritizing facile hit synthesis off-DNA. These principles should be broadly applicable, although this initial study considers a narrower scope of unbiased two-cycle libraries using only three BB classes. While this approach is readily extended to other common DEL BB classes, it does not account for stereochemistry, 3D structure,^61^ or experimentally determined BB coupling yield. Additionally, it is still unclear which areas of chemical space will be the most productive as DEL often uncovers hit compounds with unexpected binding modes.^60^ Further work is necessary to develop guidelines for three-cycle libraries or modes to identify covalent inhibitors,^62^ protein–protein interaction disruptors,^63^ or molecular degraders^64,65^ to accommodate their expanded PCPs. Additional studies comparing different dimensionality reduction techniques for DEL analysis, such as TMAP,^66^ GTM,^40^ or UMAP,^67^ are also likely to be valuable. Finally, while model library size was constrained to 192 × 192 (∼37k numerical diversity), which is at least an order of magnitude smaller than some DELs, the field is migrating toward smaller libraries with more lead-like to drug-like properties.^36,68^ These trends have accompanied experimental design innovations, enabling the detection of weaker binding events from lower cycle libraries through photoactivatable handles.^69−71^

Computational tools to understand and manage diversity are critical given the near limitless expanses of chemical space that are accessible to DEL. Estimates for the size of drug-like chemical space range from 10^23^ to 10^60^ depending on the computational method employed.^72^ Practical DEL sizes sample only a minuscule fraction of this space. One possible avenue for computational intervention is the use of theoretical database mining to identify unexplored regions of chemical space.^73^ Current methods have been employed to select BBs that narrow the PCP space of DELs,^52^ enumerate libraries,^74^ and assess DEL chemical diversity.^40^ One such modeling experiment highlighted library architecture and BB class as key drivers of library PCP and diversity, respectively.^3^ From a collection of 2497 DELs, the top 5 most diverse libraries were three-cycle libraries generated from robust coupling reactions with diverse BBs. Conversely, the least diverse libraries were generated from multiple heterocyclization reactions. While this computational assessment of diversity does not directly predict library productivity, simple library designs with diverse BBs have proven highly productive.^2,18,47^

Moving forward, improvements in DEL computational analysis tools and experimental approaches will empower new hit finding capabilities. Meta-analysis comparing the productivity of different library designs across several targets^10,47,75^ could provide valuable insights for the DEL community without requiring the disclosure of sensitive intellectual property. Such disclosures could address fundamental DEL design questions regarding optimal library size, productivity of two- vs three-cycle libraries, productivity of different BB classes, and the impact of different BB selection strategies. As DEL is arguably guided by BB diversity, new methods to synthesize novel BBs efficiently should be a high priority.^53,76,77^ Additional on-DNA reactions that utilize abundant BBs in creative ways^36^ and further development of broad-scope cross-coupling reactions^78,79^ are also likely to be highly productive. Such advances, in concert with evolving computational tools, library design schemes, selection methods, and screening strategies, will continue to hone the technology’s already indelible impact on small molecule discovery.

## 3 Methods

### 3.1 Building Block Acquisition

We acquired separate BB catalog files (in .sdf format) for purchasable Fmoc-protected amino acid BBs, primary amine BBs, and carboxylic acid BBs directly through correspondence with a representative at Enamine. Updated BB lists can also be downloaded by creating an account on the Enamine web site or through the database search feature on DataWarrior (V5.5.0).^80^ Each catalog file contains information regarding basic PCPs, cost, IUPAC name, catalog information, and RDKit molecule rendering for BBs. We provide the exact raw structure files we used on our GitHub, denoted with the file name following the format: “[functional group]_stock.sdf”.

### 3.2 Building Block Truncation

For each Enamine BB stock file, we used the OELibraryGen function from OpenEye’s OEChem module (v.2021.1.1)^81^ to generate truncated versions of each BB via reaction SMIRKS. For Fmoc-protected amino acid BBs, we replaced the—Fmoc, –NH_2_, and –COOH groups with –H. For amine BBs, we replaced –NH_2_ groups with –H. Lastly, for carboxylic acid BBs, we replaced –COOH groups with –H (Figure S1). The SMIRKS patterns and associated file preparation scripts can be found on our GitHub.

### 3.3 Building Block Filtering

We applied a SMIRKS filter to remove BBs containing secondary amines, secondary amino acids, and aniline nitrogens. To filter BBs by cost, we used pandas (v.1.2.1)^82^ to write queries based on our established cost thresholds. For PCP filtering, we calculated the relevant properties for each BB before writing queries to filter based on RO3 (MW < 300, cLog *P* ≤ 3, HBD ≤ 3, HBA ≤ 3) or RO5 (MW < 500, cLog *P* < 5, HBD ≤5, HBA ≤ 10) specifications. We exported files containing the canonical SMILES, Enamine ID, cost, and PCP of each BB as well as the canonical SMILES of its corresponding truncate as .csv files for further analysis. We provide these cleaned structure files in our GitHub and denote each file as “[functional group]_df.csv”.

### 3.4 Similarity Calculation

We used RDKit (v.2020.09.01)^83^ to calculate the 2D Tanimoto similarity among all truncates. We used the canonical SMILES for each truncate to generate an RDKit molecule object and transformed the molecule into a Morgan fingerprint with radius of 3 bonds and 2048 bits. For each truncate, we calculated its similarity to all other truncates, yielding an all-by-all similarity matrix. We provide a script for this procedure on our GitHub.textbfChemical space projections. To visualize the coverage of chemical space of different truncate selections, we used a dimensionality reduction technique known as UMAP (v.0.5.3).^28^ UMAP assigns coordinates to each truncate based on the truncate’s chemical distance to other truncates. We converted the calculated all-by-all similarity matrix into an all-by-all distance matrix and input this into the UMAP algorithm. Here, we define chemical distance by subtracting the 2D Tanimoto similarity from 1, leading to distance values ranging from 0 (very similar) to 1 (very dissimilar). The resultant output of UMAP was a set of 2D coordinates for each truncate, where chemically similar BBs were closer together in the UMAP projection and more dissimilar BBs were further away from each other, providing a depiction of the chemical space. We combined these projections with a Gaussian kernel density estimate from SciPy (v.1.5.3) to illustrate the coverage of the chemical space in this work.

### 3.5 Building Block Selection Strategies

We investigated three different BB selection strategies in this work: random, diversity, and uniform. For random selection, we used pandas (v.1.2.1) to randomly sample a specified number of BBs. In diversity selection, we used the MinMaxPicker function from RDKit to select a maximally diverse set of compounds given an initial seed and a specified number of compounds to select. The algorithm performs similarity calculations based on the underlying Morgan fingerprints. Lastly, in uniform selection, we sample from the density of the UMAP projection. In this final selection strategy, we establish a minimum distance threshold in the UMAP space and, given an initial truncate, identify truncates that are at least the established distance away in UMAP space.

### 3.6 Library Enumeration and Analysis

Once we had selected a set of BBs for each library cycle, we performed a library enumeration using the OELibraryGen function from OpenEye’s OEChem module (v.2021.1.1).^81^ In this work, we limited our analysis to two-cycle libraries where BBs in cycle 1 contain an amine group and BBs in cycle 2 contain a carboxylic acid group. Thus, our only “BB-linking” reaction was amide formation, which we represented as a SMIRKS reaction string. Once we enumerated all library products, we calculated various PCPs using OpenEye’s OEMolProp module (v.2021.1.1).^81^

## Data Availability

All original data files and code for this analysis are made publicly available on GitHub at https://github.com/MobleyLab/DEL_BB_design.

## Supporting Information Available

BB truncation, overlap between full BB truncates, overlap between truncates subset by cost, overlap between truncates subset by MW, and selection strategies for selecting carboxylic acids (PDF)

## Abbreviations

DEL: DNA-encoded library
BB: building block
PCP: physicochemical property
HTS: high-throughput screening
HBD: hydrogen bond donor
HBA: hydrogen bond acceptor
