# Supplementary material for: Building Block-Centric Approach to DNA-Encoded Library Design
Source: J Chem Inf Model. 2024 Jun 11;64(12):4661–72. doi: 10.1021/acs.jcim.4c00232 (PMC11200258; doi:10.1021/acs.jcim.4c00232)
Supplement: Supplementary file 1 — ci4c00232_si_001.pdf [file ci4c00232_si_001.pdf]

Supporting Information:

A Building Block Centric Approach to DNA-Encoded Library Design

Patrick R. Fitzgerald,[†] Anjali Dixit,[‡] Chris Zhang,[¶] David L. Mobley,[‡,¶] and Brian M. Paegel[*,‡,¶]

†*Skaggs Doctoral Program in the Chemical and Biological Sciences, Scripps Research, La Jolla, California 92037, United States*

‡*Department of Pharmaceutical Sciences, University of California, Irvine, California 92697, United States*

¶*Department of Chemistry, University of California, Irvine, California 92697, United States*

E-mail: bpaegel@uci.edu

Supporting Tables

Table S1. Computational building block truncation. Amino acids were transformed by replacing -COOH and -NFmoc functionality with -H, amines were transformed by replacing -NH_2 with -H, and carboxylic acids were transformed by replacing -COOH with -H to give ”truncates” discussed in this work. Further reduction of these truncates yields their corresponding Bemis-Murcko scaffolds.

	**Building block**	**Truncate**	**Scaffold**
**1° Amine**	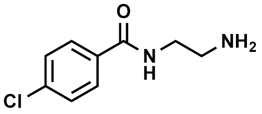	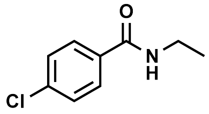	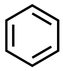
**Fmoc-Amino acid**	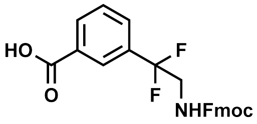	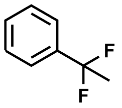	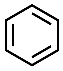
**Carboxylic acid**	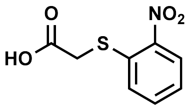	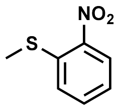	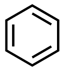

Supporting Figures

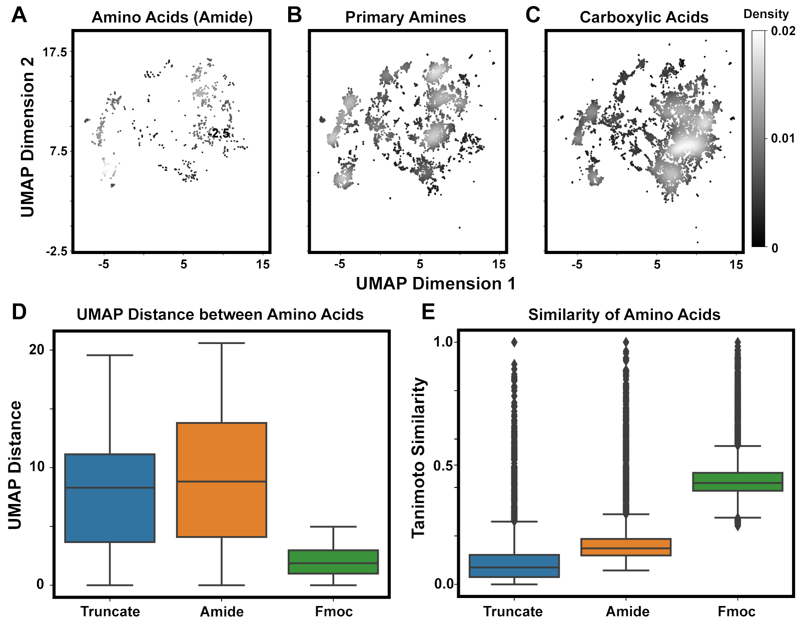

Figure S1. UMAP analysis of Fmoc-AA, primary amine, and carboxylic acid BBs from Enamine without truncation. Density plots arranged by chemical similarity are compared for **(A)** Fmoc-amino acids ($CONH_2$-R-NH_2), **(B)** primary amine (R-NH_2), and **(C)** carboxylic acids (R-$CONH_2$). Grayscale intensity denotes the probability density of points. **(D)** A box and whisker plot is generated to summarize the density of UMAP space when Fmoc-AA BBs are input as their corresponding truncates, amide representations, or fully intact structures. **(E)** A box and whisker plot is generated to summarize the Tanimoto-similarity scores for Fmoc-AA BBs are input as their corresponding truncates, amide representations, or fully intact structures.

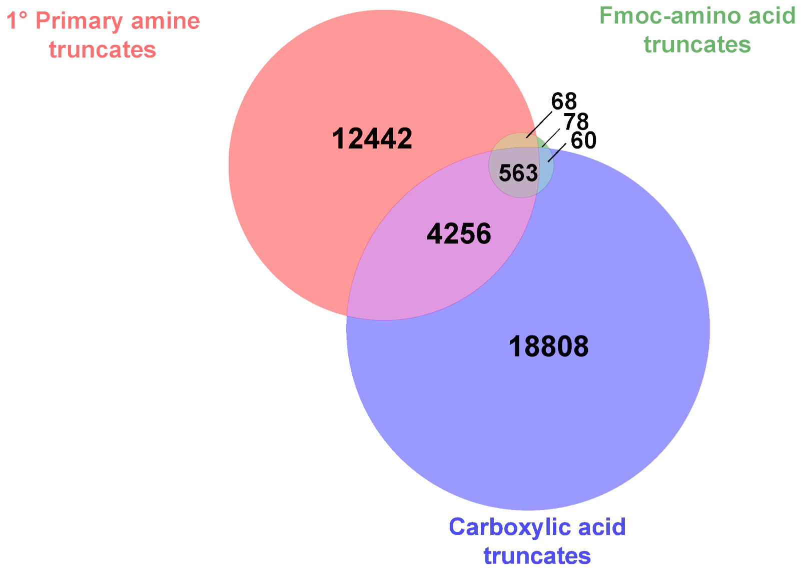

Figure S2. Venn diagram of BB truncate overlaps for Fmoc-AAs, primary amines, and carboxylic acids.

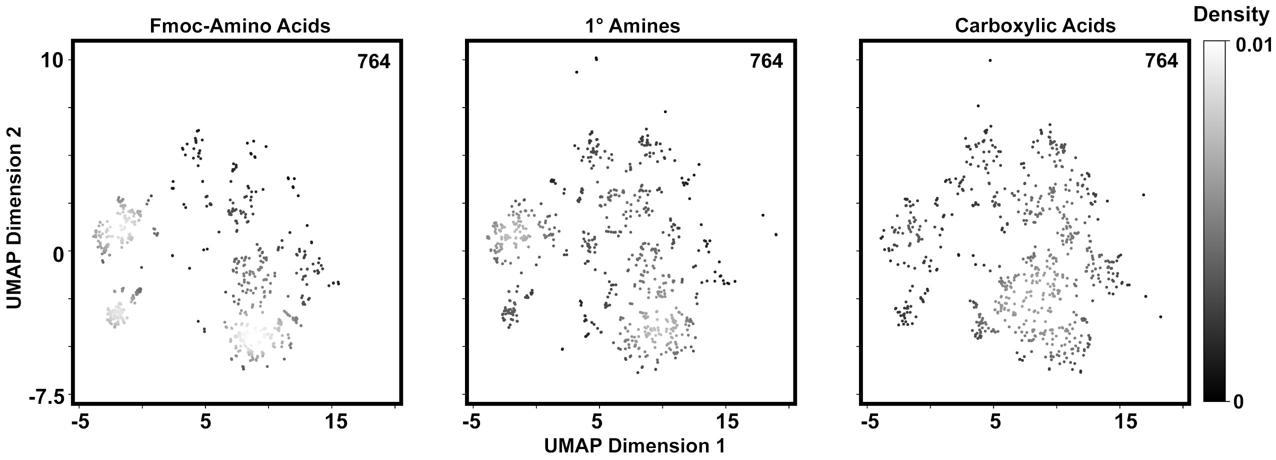

Figure S3. Comparison of Fmoc-AA, primary amine and carboxylic acid UMAP coverage following equal sampling (n = 764).

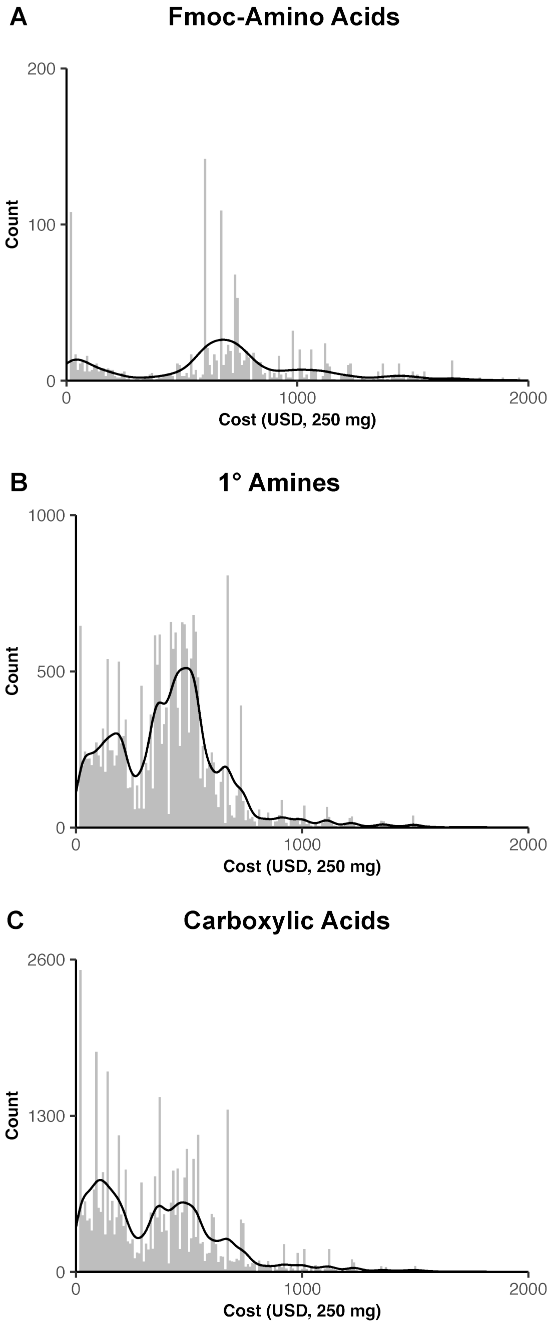

Figure S4. Cost distributions for (**A**) Fmoc-AA, (**B**) primary amine, and (**C**) carboxylic acid BB sets. BBs were binned by cost ($10 / 250 mg), and plotted along with a corresponding density trace.

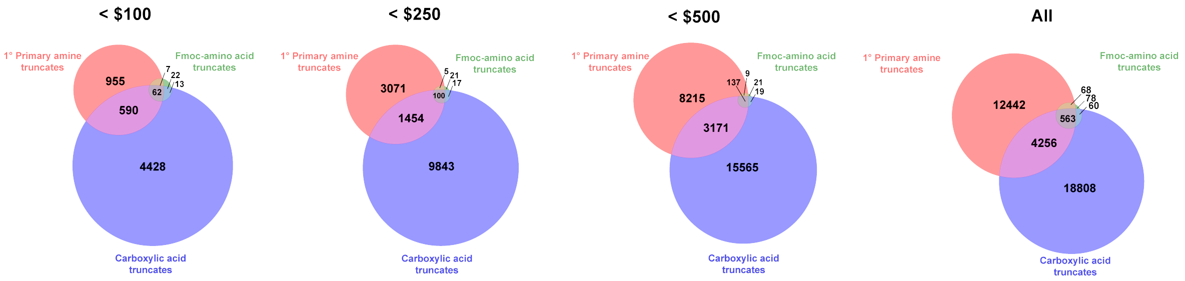

Figure S5. Venn diagrams of BB truncate overlaps subset by cost.

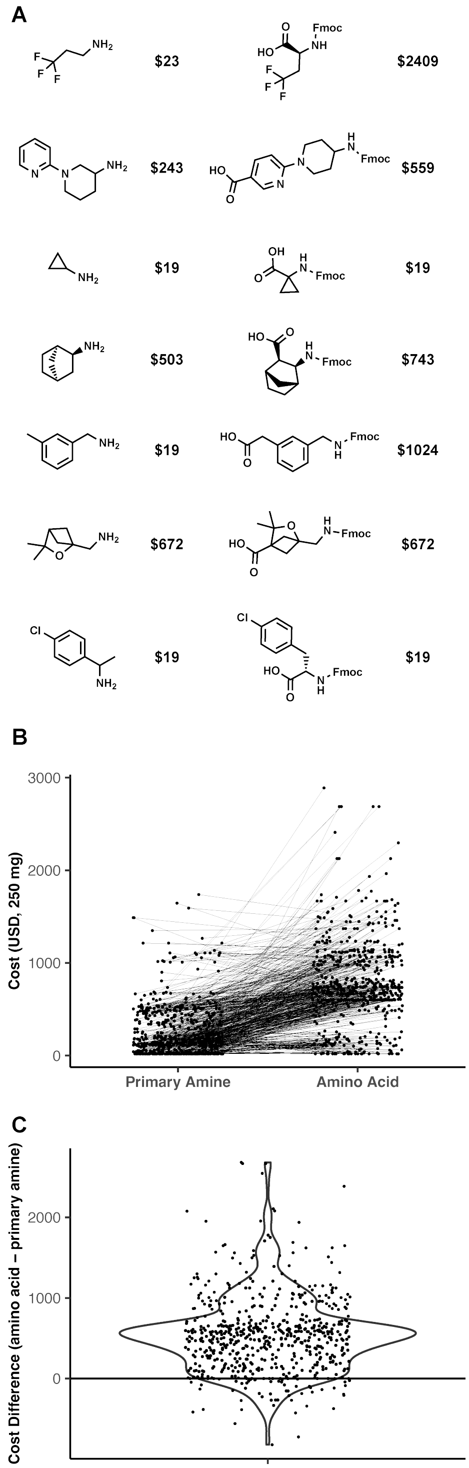

Figure S6. Cost comparison between matched primary amine and Fmoc-AA BBs. **(A)** Selected examples of paired compounds and associated costs (250 mg). **(B)** Primary amine and Fmoc-AA BBs are plotted by cost, with lines indicating compound pairs. **(C)** Data in A are reduced by plotting the difference in the cost of the pair (Fmoc-AA cost - primary amine cost). Data points are jittered to avoid overplotting and overlayed with a violin plot indicating relative density.

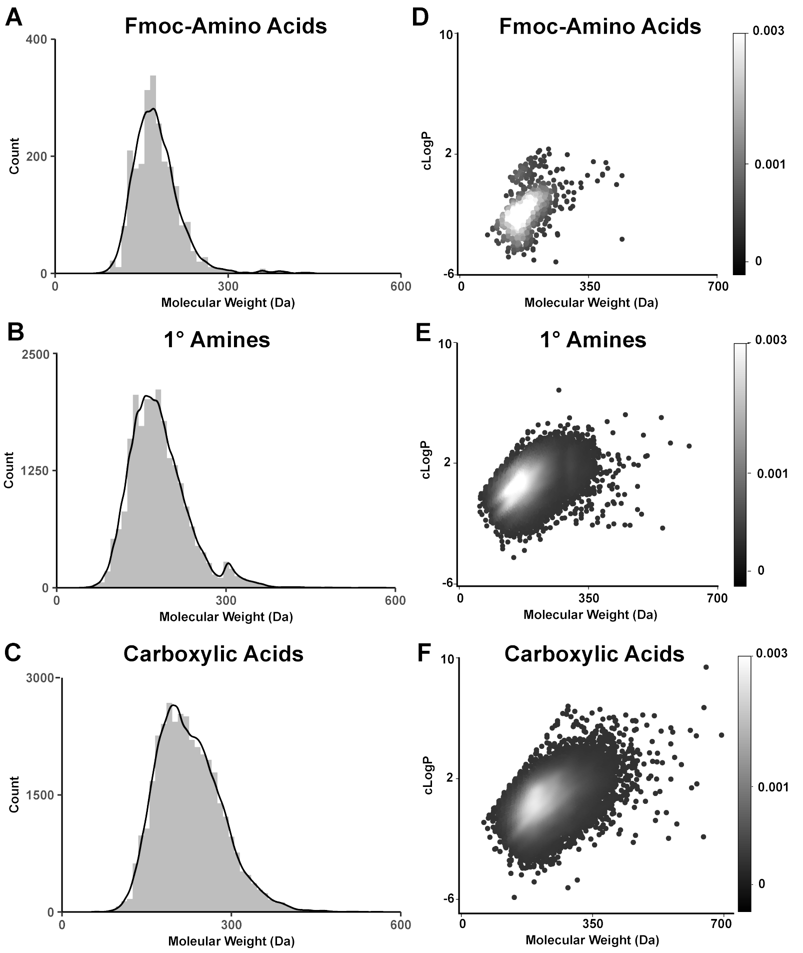

Figure S7. MW and cLogP distributions for Fmoc-AA, primary amine, and carboxylic acid BB sets. **(A-C)** BBs were binned by MW (10 Da), and plotted with a corresponding density trace. **(D-F)** MW was calculated for BBs (Fmoc removed for AAs) and plotted against predicted cLogP. Density of points is indicated by grayscale.

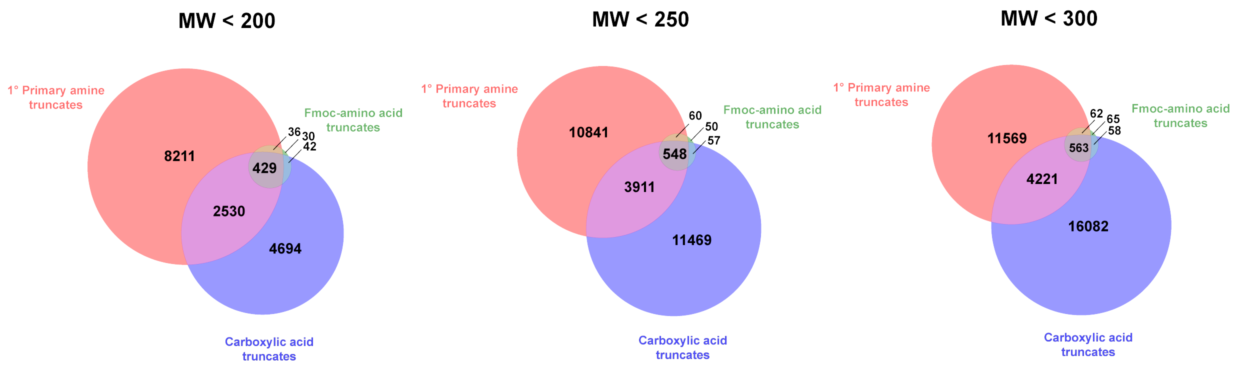

Figure S8. Venn diagrams of BB truncate overlaps subset by MW filtering (MW $\leq$ 200, 250, or 300 Da).

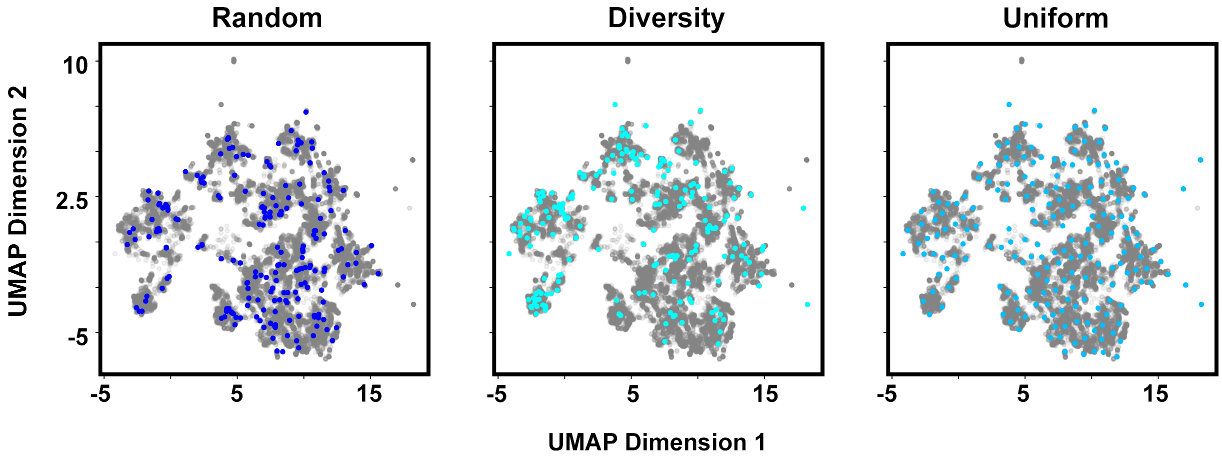

Figure S9. Illustration of UMAP coverage from random, diversity-based, and uniform selections of 192 carboxylic acids.

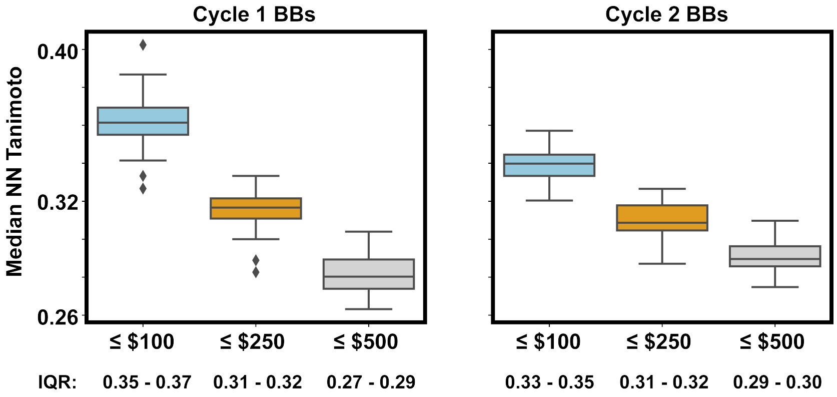

Figure S10. Comparison of chemical similarity for BBs stratified by cost. (**A**) Cycle 1 primary amine and (**B**) Cycle 2 carboxylic acid BB sets are randomly sampled (192 BBs/sampling) at variable price cutoffs ($\leq$ \$100, $\leq$ \$250, $\leq$ \$500 / 250 mg) iteratively (n = 50). Average nearest neighbor scores are calculated for each sampling and plotted as a box and whisker plot. The interquartile range (IQR) of NN Tanimoto scores for each cost filter are indicated.

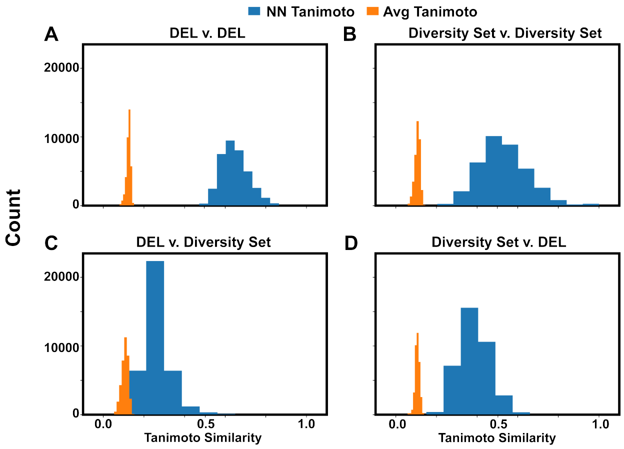

Figure S11. Comparisons within or between an enumerated library and an Enamine Lead-like set. A 192 × 192 primary amine × carboxylic acid DEL is enumerated (random BB-sampling). The all × all 2D-Tanimoto similarity matrix is generated using the enumerated DEL (n = 36,864) and a downsampling of an Enamine lead-like commercial catalog (”Diversity set”, n = 36,864). Summaries of (**A**) DEL compound intralibrary similarity, (**B**) Enamine lead-like set intralibrary similarity, (**C**) DEL vs Enamine library, and (**D**) Enamine library vs DEL similarity are plotted as histograms, with nearest neighbor scores in blue, and average Tanimoto scores in orange.
